# Assessment of the Brain's Macro- and Micro-Circulatory Blood Flow Responses to CO_2_ via Transfer Function Analysis

**DOI:** 10.3389/fphys.2016.00162

**Published:** 2016-05-09

**Authors:** Martin W.-D. Müller, Mareike Österreich, Andreas Müller, John Lygeros

**Affiliations:** ^1^Department of Neurology and Neurorehabilitation, Kantonsspital LucerneLucerne, Switzerland; ^2^Automatic Control Laboratory, ETH ZurichZurich, Switzerland

**Keywords:** cerebral autoregulation, cerebral blood flow, transcranial Doppler ultrasound, near-infrared spectroscopy, transfer function

## Abstract

**Objectives:** At present, there is no standard bedside method for assessing cerebral autoregulation (CA) with high temporal resolution. We combined the two methods most commonly used for this purpose, transcranial Doppler sonography (TCD, macro-circulation level), and near-infrared spectroscopy (NIRS, micro-circulation level), in an attempt to identify the most promising approach.

**Methods:** In eight healthy subjects (5 women; mean age, 38 ± 10 years), CA disturbance was achieved by adding carbon dioxide (CO_2_) to the breathing air. We simultaneously recorded end-tidal CO_2_ (ETCO_2_), blood pressure (BP; non-invasively at the fingertip), and cerebral blood flow velocity (CBFV) in both middle cerebral arteries using TCD and determined oxygenated and deoxygenated hemoglobin levels using NIRS. For the analysis, we used transfer function calculations in the low-frequency band (0.07–0.15 Hz) to compare BP–CBFV, BP–oxygenated hemoglobin (OxHb), BP–tissue oxygenation index (TOI), CBFV–OxHb, and CBFV–TOI.

**Results:** ETCO_2_ increased from 37 ± 2 to 44 ± 3 mmHg. The CO_2_-induced CBFV increase significantly correlated with the OxHb increase (*R*^2^ = 0.526, *p* < 0.001). Compared with baseline, the mean CO_2_ administration phase shift (in radians) significantly increased (*p* < 0.005) from –0.67 ± 0.20 to –0.51 ± 0.25 in the BP–CBFV system, and decreased from 1.21 ± 0.81 to −0.05 ± 0.91 in the CBFV–OxHb system, and from 0.94 ± 1.22 to −0.24 ± 1.0 in the CBFV–TOI system; no change was observed for BP–OxHb (0.38 ± 1.17 to 0.41 ± 1.42). Gain changed significantly only in the BP–CBFV system. The correlation between the ETCO_2_ change and phase change was higher in the CBFV–OxHb system [*r* = −0.60; 95% confidence interval (CI): −0.16, −0.84; *p* < 0.01] than in the BP–CBFV system (*r* = 0.52; 95% CI: 0.03, 0.08; *p* < 0.05).

**Conclusion:** The transfer function characterizes the blood flow transition from macro- to micro-circulation by time delay only. The CBFV–OxHb system response with a broader phase shift distribution offers the prospect of a more detailed grading of CA responses. Whether this is of clinical relevance needs further studies in different patient populations.

## Introduction

Cerebral autoregulation (CA) describes the ability of the cerebrovascular system to provide a continuous steady-state blood supply to the brain over a wide range of blood pressure (BP) levels. Cerebral perfusion exhibits a linear relationship with BP beyond the CA maintenance range (Kontos et al., 1978; Harper et al., 1984). Low BP leads to low cerebral perfusion and may result in ischemia (Ringelstein et al., 1988; Kleiser and Widder, 1992). The methods used at present to confirm CA integrity have revealed that patients with a disrupted or decreased CA have a greater risk of a poorer outcome compared with those with an intact CA (Kleiser and Widder, 1992; Müller et al., 1995, 2003; Müller and Schimrigk, 1996; Reinhard et al., 2003). The current methods widely used for CA analysis include high time resolution methods, such as transcranial Doppler sonography (TCD), near-infrared spectroscopy (NIRS), or laser speckle imaging; these methods are non-invasive and can frequently be repeated if necessary over time periods of several hours (Zhang et al., 1998; Panerai et al., 1999; Terborg et al., 2000; Murkin and Arango, 2009; Zweifel et al., 2010; Cooper et al., 2011; Taussky et al., 2012; Hecht et al., 2013; Müller and Österreich, 2014; Nielsen, 2014).

TCD measures blood flow velocity in large cerebral arteries. The actual measured blood flow velocity depends on several factors, mainly on the BP gradient across the vessel bed and on the vessel diameter. Metabolic factors such as partial pressure of carbon dioxide (pCO2), mental activity, or [H^+^] concentration in the brain tissue may additionally affect vessel diameter and velocity. The measured velocity represents the actual brain demands and corresponds closely to the cerebral blood flow (CBF) when vessel diameter does not change considerably. Insofar TCD data represent CBF in the brain's macro-circulation. NIRS investigates the cortical microangiopathic capillary vessel bed. The mechanisms of CA transform macroangiopathic blood flow to microangiopathic capillary flow, thus linking the processes.

CA is mostly characterized by the relationship between BP and macro-circulatory CBF or its velocity (CBFV). The micro-circulatory CBF can be considered blood flow after CA mechanisms have regulated the macro-circulatory input CBF. The interaction between the macro- and micro-circulatory levels has been rarely investigated (Reinhard et al., 2006; Phillip et al., 2012). Herein, we used high temporal resolution methods that would particularly allow assessment of the dynamic aspects of such interactions. This approach could provide additional insights into autoregulatory processes and might facilitate the future development of mathematical models for CA status predictions. Such a predictability of CA would be helpful in diseases (such as traumatic brain injury, subarachnoid hemorrhage, stroke) in which it is known that secondary ischemic events due to CA failure can follow the initial brain damage and worsen patient's outcome. In order to explore whether one of the two methods or a combination of both is more suitable for this purpose, we applied both methods simultaneously.

## Methods

This study was approved by the local ethics committee. It follows the Declaration of Helsinki and good clinical practice standards. All subjects provided written informed consent. Eight healthy volunteers (5 women; mean age, 38 ± 10 years) were investigated while in the supine position with the head elevated ~30°. Each investigation was performed in the late morning. After mounting all probes and adapting the subject to the experimental setting, baseline values were recorded over a minimum period of 10 min. After that, a CO_2_ enriched air mixture (7% CO2, 93% Oxygen) was administered until a clear CBFV increase was identified. CO_2_ administration was then maintained for 10 min.

To assess CBFV, we used TCD (MultidopX, DWL; Compumedics, Sipplingen, Germany) with a 2-MHz probe to insonate the bilateral middle cerebral arteries (MCAs) through the temporal skull; the probes were fixed using a head holder provided by the manufacturer. The MCAs were identified according to commonly used criteria. BP was measured non-invasively by finger plethysmography (Finometer Pro; Finapres Medical Systems, Amsterdam, The Netherlands). A cerebrovascular resistance (CVR) index was calculated by BP/CBFV. To assess the micro-circulation using NIRS, we used the NIRO-200NX device (Hamamatsu Photonics, Herrsching, Germany). This NIRS device emits infrared light at three frequencies (735, 810, 850 nm); the backscattered light exhibits different intensities after absorption by oxygenated and deoxygenated hemoglobin. Differences in light intensities between the emitted and backscattered light correlate with the concentrations of oxygenated and deoxygenated hemoglobin in the brain's upper layers. We used self-adhesive NIRS probes in which the light emitting diode (LED) and detecting photodiode were fixed 3.5 or 4 cm apart. The detecting probe was placed over the frontotemporal lobe, and the emitting probe was placed on the frontal skull. The probes with the 4 cm distance between the LED and receiving diode were always placed on the right side; the other probe (with a distance of 3.5 cm between the diodes) was placed on the left side. We used both types of probes to address the potential relevance of differences in the penetration depth. After making initial adjustments to determine a baseline hemoglobin concentration, the NIRS device then provides information about changes in the hemoglobin concentrations (in μmol/L) from the baseline. Because oxyhemoglobin-derived data provides the best signal intensity for transfer function analyses (Reinhard et al., 2006; Phillip et al., 2012), we restricted our analysis to the oxyhemoglobin-derived data, namely oxygenated Hb (OxHb) and the total oxygenation index (TOI), defined as (OxHb/oxygenated + deoxygenated Hb) and reported as a percentage. The end-tidal pCO_2_ (ETCO_2_) concentration was measured using the capnograph embedded in the TCD device. To measure ETCO_2_, the small collecting tube of the capnograph was placed in one nostril. In the other nostril, a larger tube was placed through which an air mixture was added to the breathing air to inducing pCO_2_-related blood flow changes. The ETCO_2_ for each patient was reported as the mean ETCO_2_ over the total recording period.

### Data preparation

The minimum recording time was 10 min. BP, CBFV, and pCO_2_ data were collected at 100 Hz, and NIRS data were collected at 20 Hz. Data were analyzed using Matlab (2015b; MathWorks Inc., Natick, MA, USA). Data were visually inspected for artifacts. Only artifact-free data periods were used. For each subject, the recordings contained bilateral artifact-free periods of 7 min in both modalities (baseline and CO_2_ administration); therefore, a total of 16 hemispheres were analyzed. After aligning the time series with their common starting time point, each raw data time series was recollected by averaging to 1 s. The coherence and TF estimates of the phase and gain of the different time series were extracted from their respective power auto spectra or cross spectra using Welch's averaged periodogram method, with a Hanning window length of 100 s, a window overlap of 50%, and a total Fast Fourier Transformation data length of 400 s. For each subject, the coherence, phase (in radians), and gains (in cm/s/mmHg for the BP–CBFV system, in μmol/L/mmHg or %/mmHg for the BP–NIRS data system, and in μmol/L/cm/s or %/cm/s for the CBFV–NIRS data) were calculated over a frequency range of 0.02–0.40 Hz. At each frequency phase, gain and coherence are calculated. Phase indicates that the corresponding sinus waves of (e.g.,) a period length of 10 s (= 0.1 Hz) from BP and CBFV are congruent in time (phase = 0) or dissociated from each other (one is earlier or later than the other). Gain indicates how much power is transmitted from the BP wave to the CBFV wave. Coherence indicates how stable over time the phase relationship between the two waves is; 0 indicates no stability, 1 a perfect stability with then a high consistency of the calculated phase and gain values.

### Statistical analysis

All values are reported as mean ± standard deviation (SD). Pearson correlations (95% confidence interval (CI)) and paired *t*-tests were used for analysis. A *p* < 0.05 is considered to indicate a significant difference.

## Results

The mean values of the relevant physiological and TF parameters at baseline and after CO_2_ administration are listed in Table 1. Of note, the ETCO_2_ increase was accompanied by a small BP increase. CO_2_ induced CBFV increase (in % from baseline) correlated significantly with the CO_2_ induced OxHb increase [*r* = 0.72 (95% CI: 0.36, 0.89), *p* < 0.005)], as well as with the CO2 induced TOI increase [*r* = 0.69; 95% CI: 0.30, 0.80; *p* < 0.005].

**Table 1:** **Physiological variables and transfer function parameters in the 0.07–0.15 Hz range in different systems at baseline and after CO_2_ administration**.

**Variable**	**Baseline**	**After CO_2_administration**	***p*-value (<)**
BP (mmHg)	80±9	87±6	0.01
ETCO_2_ (mmHg)	37±2	44±3	0.001
CBFV (cm/s)	63±14	79±25	0.001
CVR (mmHg/cm/s)	1.23±0.23	1.10±0.25	0.002
**BP–CBFV SYSTEM**			
Coherence	0.75±0.13	0.78±0.13	n.s.
Phase (radians)	−0.67±0.20	−0.51±0.25	0.005
Gain (cm/s/mmHg)	0.63±0.22	0.73±0.25	0.05
**BP–OxHb SYSTEM**			
Phase (radians)	0.38±1.17	0.27±1.42	n.s.
Gain (μmol/L/mmHg)	0.00±0.01	0.00±0.02	n.s.
**BP–TOI SYSTEM**			
Phase (radians)	0.00±0.01	0.09±1.30	n.s.
Gain (%/mmHg)	0.00±0.02	0.00±0.03	n.s.
**CBFV–OxHb SYSTEM**			
Phase (radians)	1.21±0.81	−0.05±0.91	0.005
Gain (μmol/L/cm/s)	0.00±0.02	0.00±0.02	n.s.
**CBFV–TOI SYSTEM**			
Phase (radians)	0.94±1.22	−0.24±1.0	0.005
Gain (%/cm/s)	0.00±0.04	0.00±0.02	n.s.

**Figure 1:**
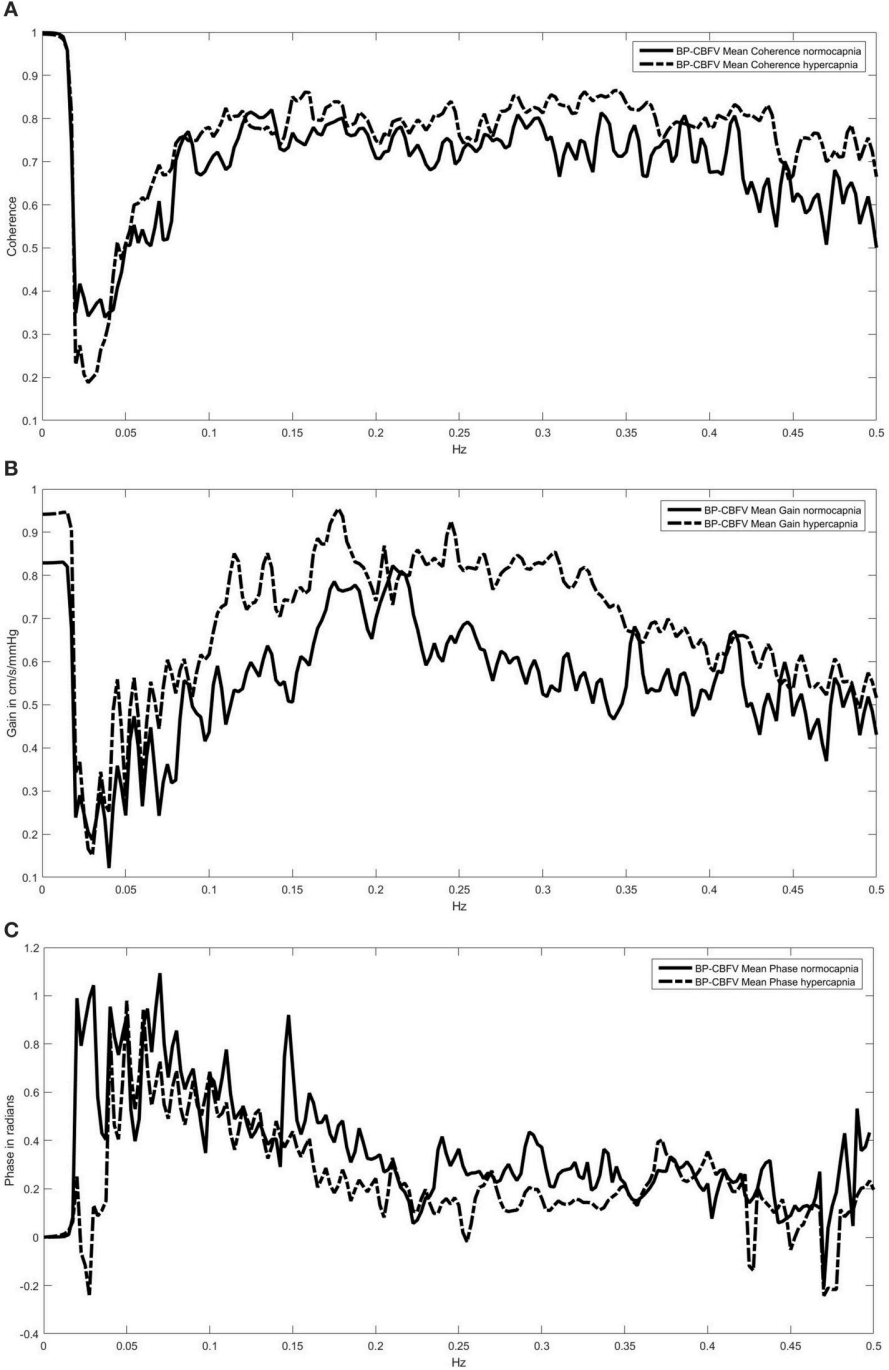
**Coherence (A) and transfer function spectra (gain B; phase shift C) of the blood pressure (BP)—cerebral blood flow velocity (CBFV) system under normo- and hypercapnia**. The high coherence indicates a highly stable phase relationship over the whole frequency range. For convience, we show in all three parts the means only and did not include the SD range of both curves.

**Figure 2:**
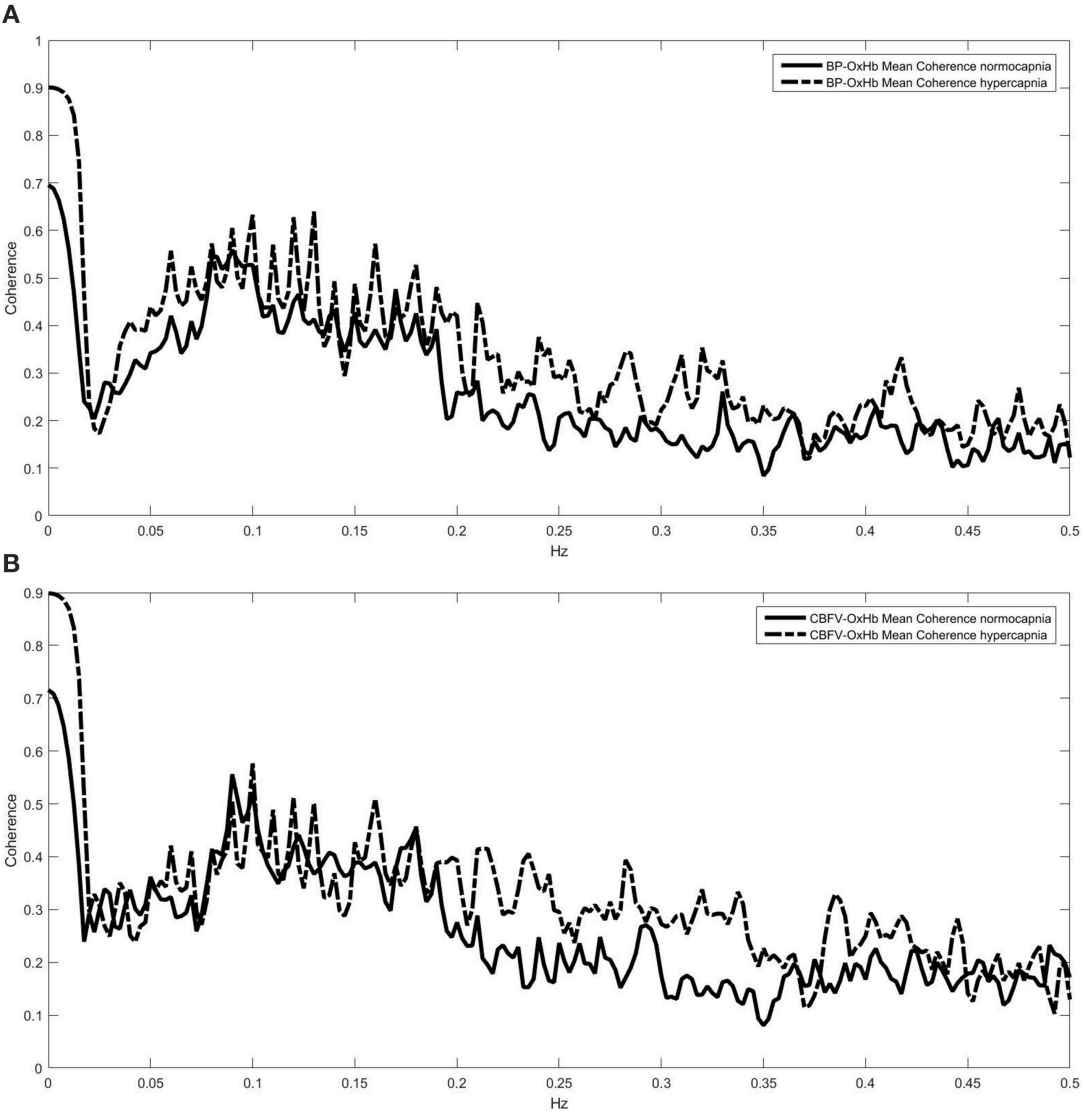
**Coherence spectra under normo- and hypercapnia for the blood pressure (BP)—oxygenated hemoglobin (OxHb, A), and the cerebral blood flow velocity (CBFV)—OxHb systems (B)**. In both systems coherence indicates that further transfer function estimations are reliably performed in the 0.05–0.20 Hz range only.

TF analysis of the BP-CBFV system (Figures 1A–C) showed a high coherence so that gain and phase estimations are valid. All Coherence analyses which included OxHb or TOI showed a relevant coherence in the frequencies between 0.05 and 0.2 Hz only (two examples are provided in Figures 2A,B). Because CA is mostly regulated in the frequency range of 0.07–0.15 Hz, we used the TF parameters of this frequency range for analysis. Only phase and gain values at a coherence ≥0.3 were considered for analysis (this level was considered to provide reliable data) (Meel-van den Abeelen et al., 2014).

Regarding the BP–CBFV relationship, CBFV led BP by –0.67 ± 0.20 radians (Table 1), which was increased to –0.51 ± 0.25 radians after CO_2_. BP initially led OxHb by a phase of 0.38 ± 1.17 and did not significantly change after CO_2_ administration. In the CBFV–OxHb system, OxHb followed CBFV by a phase shift of 1.21 ± 0.81 radians at baseline; this phase shift changed to −0.05 ± 0.91 after CO_2_ administration. CBFV–TOI exhibited similar behavior.

Compared to baseline, 3 TF variables changed significantly after CO_2_ administration, and one variable exhibited a trend. The BP–CBFV phase changes in each hemisphere correlated significantly with CO_2_ changes (*r* = 0.52; 95% CI: 0.03, 0.80; *p* < 0.05); the best correlations were obtained when phase changes were expressed in percent changes from baseline and the results of both hemispheres were subsequently averaged to yield 1 result per subject (*r* = 0.63; 95% CI: −0.13, −0.92; *p* = 0.09). The CBFV–OxHb phase change correlated with the change in CO_2_ (*r* = −0.60; 95% CI: −0.16, −0.84; *p* < 0.01) such that a greater ETCO_2_ change yielded a more negative CBFV–OxHb phase. Of note, the probe with two diodes separated at a 4 cm distance performed slightly better (*r* = −0.67; 95% CI: −0.93, 0.05; *p* = 0.06) than did the probe with diodes at a 3.5 cm distance (*r* = −0.53; 95% CI: −0.90, 0.22). The CBFV–TOI phase change did not correlate with the CO_2_ administration but exhibited a trend with BP changes (*r* = 0.44, *p* = 0.08). Remarkably, the BP–CBFV gain was the only gain variable to exhibit a change. For further interpretation of gain change the corresponding change of CVR is necessary. CO_2_ administration induced a significant CVR decrease (Table 1) but only in the BP-CBFV system the correlation between CVR change and gain change [*r* = −0.60 (95% CI: −0.16, −0.84; *p* < 0.04] reached clear significance indicating gain increases the more the more CVR decreases after CO_2_ administration.

## Discussion

In recent years, the use of TF to estimating CA via analysis of the BP–CBFV relationship clarified the dependence of results on several technical aspects (Meel-van den Abeelen et al., 2014), including how signals are averaged (beat-by-beat or raw wave, sample frequency), the window selected for FFT, and decisions regarding signals smoothing or analysis of relative values (for details, see Claassen et al., 2016). Our baseline results regarding the phase shift between BP and CBFV are in agreement with the observed spread (Table 2 and Zhang et al., 1998; Panerai et al., 1999; Müller et al., 2003; Reinhard et al., 2003). Under pathological conditions, when phase shift approaches 0° this phase shift spread narrows.

**Table 2:** **Mean phases (in angles) and time intervals between blood pressure (BP), cerebral blood flow velocity (CBFV), and oxygenated hemoglobin (OxHb)**.

**Author**	**BP–CBFV**	**BP–OxHb**	**CBFV–OxHb**
**BASELINE**			
Own results	−38°/1.05 s	21°/0.56 s	69°/1.83 s
Reinhard et al., 2006	−64°/1.78 s	23°/0.62 s	84°/2.26 s
Phillip et al., 2012	−57°/1.53 s	15°/0.40 s	72°/1.94 s
**PATHOLOGICAL CONDITION**			
Own results: CO_2_ administration	−29°/0.78 s	15°/0.41 s	0°/0 s
Reinhard et al., 2006: Occlusive carotid artery disease	−34°/0.91 s	29°/0.78 s	65°/1.75 s

*For comparisons to literature reports in which TCD and NIRS were simultaneously recorded, our results were based on a period of 10 s*.

Under the two evaluated pathological conditions (Table 2), the most striking difference between our results and those of Reinhard et al. (2006) is the finding that the phase shift between CBFV and OxHb was 0° after CO_2_ administration but is 65° in a group of patients with occlusive artery disease. One explanation for this discrepancy might be that CO_2_ diminishes CA rapidly, whereas the most chronic process associated with carotid artery occlusive disease allows the development of a graduated cerebrovascular response that depends on blood flow through a stenotic vessel and the development of sufficient collateral blood flow (Müller and Schimrigk, 1996; Reinhard et al., 2003). Compared to the baseline phase shift of 84° between CBFV and OxHb, the phase shift of 64° indicates a reduced but not abolished CA. Regarding the time in seconds, CO_2_ led to a direct transformation of macro-circulatory to the micro-circulatory blood flow, whereas this process remained under CA control in the occlusive vessel group.

As shown by Reinhard et al. (2006), CBFV is ahead of BP and has to be signed mathematically by a negative phase shift when BP is the reference. From there on, OxHb follows CBFV with positive phase shifts. Therefore, the cerebrovascular response to CO_2_ demonstrated that the (negative) phase between BP and CBFV shifts toward 0° increases with increasing CO_2_ (Zhang et al., 1998; Panerai et al., 1999; Müller et al., 2003) while the positive phase shifts of the CBFV–OxHb system are reduced until 0. Regardless of which parameter is used, with the present interpretation of the TF model of CA a phase shift of 0 or near zero indicates that the depending flow (CBFV or OxHb) follows the driving force (BP or CBFV) without delay which is equivalent to an abolished CA. Such a situation is pathophysiologically considered a highly risky condition of the brain because a (therapeutically) unanswered BP drop can be followed by cerebral ischemia. Our observation is that the response distribution was more widely spread in the CBFV–OxHb system than in the BP-CBFV system, as indicated by the range of their respective mean phase changes (BP–CBFV: 0.67 to 0.51 radians; CBFV–OxHb: 1.21 to −0.05 radians). The correlation analysis results are slightly in favor of the CBFV-OxHb system, so one could suggest that this system can graduate CA disturbances more precisely. One example of its suggested advantage could be that the flow-to-flow model displayed CA failure (0 radians) at times when the pressure-to-flow system indicated some autoregulation still present (0.51 radians). Because the flow-to-flow system reflects closer CBF changes than the pressure-to-flow system, the flow-to-flow systems should be further evaluated. One question is whether the broader phase response distribution is indeed more precisely than the BP-flow system to graduate CA disturbances. A second question is whether our suggestions can be applied to diseases with arterial diameter changes (e.g., subarachnoid hemorrhage); initial experience using TOI seems promising (Zweifel et al., 2010).

Although the CBFV-TOI system demonstrated a clear CO_2_ induced change, it was a surprise to recognize that this change didn't correlate with ETCO_2_ but with BP changes. TOI is calculated from both OxHb and deoxygenated hemoglobin via total hemoglobin concentration. Deoxygenated hemoglobin is mostly present in the venous part of the micro-circulation. Speculatively, it can be assumed that the venous system is regulated by other additional mechanisms apart from BP.

Regarding gain changes, we did not observe a gain transfer from macro- to micro-circulation in the flow-to-flow systems; a significant gain change was only present in the BP–CBFV system. Similar results were described by Phillip et al. (2012). Gain is considered a function of vasculature and shows an inverse relationship to cerebrovascular resistance (Aaslid et al., 1989; Tiecks et al., 1995; Serrador et al., 2005; Zhang et al., 2009). Ideally, the product of gain and CVR remains approximately constant (Zhang et al., 2009). CVR is considered a myogenic function of the smooth vessel cells (Aaslid et al., 1989; Schubert and Mulvany, 1999; Zhang et al., 2009). In an animal experiment Kolb et al. (2007) inhibited the myogenic action by Ca^2+^ channel antagonists; in the animals (rats) with Ca^2+^ channel inhibition the experimentally induced gain decrease was significantly less than the one in the control condition without Ca^2+^ channel inhibition. Recent similar observations in human beings (Tzeng et al., 2011; Tan et al., 2013) strengthen the assumption that gain is also a myogenic function. That means on the other hand that the flow-to-flow systems do not describe the pressure dependent autoregulatory processes completely.

Our study had some limitations. Apart from the technical limits as mentioned above, we address two others. First, we used probes in which diodes were separated by different distances. As indicated by our correlation analysis of CBFV–OxHb phase changes, probes in which diodes were separated by a 4-cm distance yielded better results than those in which diodes were separated by a 3.5-cm distance. This phenomenon was described similarly for a probe containing diodes separated by a 3-cm distance (Murkin and Arango, 2009). A shorter distance between the two diodes causes less brain parenchyma and more skin to be involved in measured changes in oxygenation. Therefore, one might speculate that our overall results when using only probes with 4-cm distances could be closer to the findings reported by Reinhard et al. (2006) and Phillip et al. (2012).

Second, we reported results within a particular frequency range (0.07–0.15 Hz), whereas Reinhard et al. (2006) and Phillip et al. (2012) reported results obtained at a distinct frequency of 0.1 Hz. This difference may have led to a systematic failure in our results by including time periods < 10 s in our analysis, which resulted in a shorter overall time delay/phase shift. Moreover, the methodical differences in performing TF, described in the first paragraph of this section, could lead to differences in the absolute phase shift values. Nevertheless, our overall results exhibit the same apparent direction as those of Reinhard et al. (2006) and Phillip et al. (2012).

## Conclusion

We assessed CA by two approaches: the BP-to-flow, and the flow-to-flow transition. The flow –to-flow system is characterized by phase (time delay) changes only; the BP-CBFV system reacts additionally with gain changes, which correspond most likely to a myogenic reaction. Using correlation analysis between ETCO_2_ changes and the respective phase changes, a descriptive interpretation indicates that the flow-to-flow system CBFV-OxHb may allow a more detailed grading of CA than the BP-CBFV system by providing a broader phase shift response distribution. As an example we found, that CA is already abolished in the flow-to-flow system when the BP-to-flow system did show autoregulation; in this situation the risk of further brain damage was more clearly displayed by the flow-to-flow system than by the BP-to-flow system. However, which of both approaches is the physiologically and, hence, the clinically more relevant needs further studies in different patient populations.

## Author contributions

MM: data and statistical analysis, study design, writing, intellectual content. MÖ: data collection, study design. AM: data analysis, intellectual content. JL: data interpretation, writing, intellectual content.

### Conflict of interest statement

The authors declare that the research was conducted in the absence of any commercial or financial relationships that could be construed as a potential conflict of interest.
